# Innovative oral hygiene strategies for children with autism spectrum disorder: a gamified app-based intervention

**DOI:** 10.1007/s40368-025-01115-5

**Published:** 2025-10-01

**Authors:** Z. Yerkibayeva, G. Yermukhanova, K. Saduakasova, Y. Menchisheva, K. Rakhimov, Z. Abu, D. Abdukalikova, N. Bainazarova, A. Abutalipova

**Affiliations:** 1 Asfendiyarov Kazakh National Medical University, Almaty, Kazakhstan; 2 Al-Farabi Kazakh National University, Almaty, Kazakhstan; 3 King’s College London, London, United Kingdom; 4 Asfendiyarov Kazakh National Medical University, Almaty, Kazakhstan; 5 Kazakhstan Medical University, Almaty, Kazakhstan; 6 Kazakh-Russian Medical University, Almaty, Kazakhstan

**Keywords:** Autism spectrum disorder, Mobile applications, Educational technology, Mobile health, Hygiene, Child development

## Abstract

**Purpose:**

This study aimed to evaluate the effectiveness of the “Marzhan Tis” mobile application in improving oral hygiene practices among children with autism spectrum disorder (ASD) and to examine its usability and correlation with learning success.

**Methods:**

A prospective, non-randomized cohort study was conducted with 90 children aged 3–18 years diagnosed with Level 1 ASD from rehabilitation centers in Almaty, Kazakhstan. Participants were divided into an intervention group (IG), which used the app for 1 month, and a control group (CG), which received standard oral hygiene instructions. Oral hygiene was assessed using the Simplified Oral Hygiene Index (OHI-S), approximal plaque index (API), and papillary–marginal–alveolar index (PMA). Adherence was measured using the modified Oral Hygiene Assessment Scale (OHAS-10), and a structured 15-item caregiver questionnaire evaluated behavioral changes and app usability. Statistical analysis included the Shapiro–Wilk test for normality, Mann-Whitney *U* and Wilcoxon signed-rank tests for between and within-group comparisons, Chi-square tests for categorical variables, and Pearson correlation for usability–learning associations.

**Results:**

Significant improvements were observed in the IG for all oral hygiene indices (OHI-S reduction: 28–52%; API and PMA improvement > 50%, *p* < 0.05). Oral hygiene adherence increased by 18.8% (*p* = 0.001). Independence in brushing improved in 85% of IG children (*χ*^2^ = 28.7, *p* = 0.001). A strong correlation was found between app usability and learning outcomes (*r* = 0.65, *p* = 0.01).

**Conclusion:**

The “Marzhan Tis” mobile app effectively improved oral hygiene skills and independence in children with ASD Level 1. Its usability and gamified features support its integration into pediatric oral health interventions.

## Introduction

Children with autism spectrum disorder (ASD) face significant disparities in oral health compared to their neurotypical peers. Studies consistently report higher rates of dental caries and poorer oral hygiene indices in this population, attributed to a range of behavioral, sensory, and systemic challenges (Como et al. 2022). Globally, the prevalence of ASD is estimated to be approximately 1 in 100 children, with some regions reporting rates as high as 1 in 54 (Sherriff et al. 2023). These rising figures underscore the increasing likelihood that dental professionals will care for children with ASD, yet many providers report inadequate training in managing children’s complex needs (Santosh et al. 2021).

Barriers to oral care in children with ASD are multifactorial. Sensory sensitivities, such as aversions to bright lights, loud sounds, and tactile stimuli, can make dental visits distressing. Communication difficulties and behavioral rigidity further complicate home care routines like tooth brushing. Additionally, systemic inequities—such as insurance discrimination and provider bias—exacerbate oral health disparities, particularly in underserved populations (Como et al. 2022; Carli et al. 2022). As a result, children with ASD often rely heavily on caregivers for oral hygiene maintenance and may present with advanced periodontal disease when care is delayed.

To address these challenges, researchers and clinicians have developed various adaptive strategies. These include behavioral interventions like the “Tell–Show–Do” method, visual supports (PECS, social stories, video modeling), and environmental modifications such as sensory-adapted dental environments (SADEs) (Dontsova et al. 2021; Dental problems in children with autism 2024; Chen et al. 2021; Alshatrat et al. 2021; Zhou et al. 2020; Blomqvist et al. 2015; Tan et al. 2024; Kraus et al. 2025; Piraneh et al. 2022). Evidence shows these tools can significantly reduce plaque accumulation by 56% (*p* = 0.001) and improve behavioral compliance in children with ASD (Alshatrat et al. 2021). Furthermore, structured learning strategies and interactive technologies have been found effective in enhancing self-care skills (Dontsova et al. 2021; Dental problems in children with autism 2024; Chen et al. 2021). Fallea et al. demonstrated that SADEs significantly increased treatment success from 20 to 68%) (Fallea et al. 2022). Balian et al. further emphasized the utility of visual aids, including PECS, videos, and social stories, in reducing plaque by 20–50% and improving cooperation by 60–80% (Balian et al. 2021). In particular, mobile health applications represent a promising avenue for supporting and reinforcing oral hygiene routines at home.

Gamified digital tools, which use game design elements to motivate behavior change, have gained popularity in pediatric health promotion. These apps often incorporate rewards, visual prompts, and interactive instructions to engage users. However, a 2021 review of 562 mobile applications for dental caries prevention found that only 7.1% met quality criteria, and none were specifically tailored to young children with ASD (Chen et al. 2021). This highlights a critical gap in both product development and targeted research.

In countries such as the USA, Sweden, and Japan, greater access to specialized dental services and caregiver training has been associated with better oral health outcomes in children with ASD (Zhou et al. 2020; Blomqvist et al. 2015; Zerman et al. 2022). Conversely, in regions like Pakistan, Egypt, and the UAE, higher caries rates persist due to limited services and socioeconomic constraints (Tan et al. 2024). In Kazakhstan, ASD prevalence has increased significantly from 0.8 to 12.7 per 100,000 over the past 13 years (Zhumadilova et al. 2021). Yet no published data exist on the oral health status of affected children, caregiver practices, or the use of digital interventions. This lack of region-specific evidence hinders the development of culturally appropriate, scalable strategies to improve oral health outcomes in this growing population.

To address this gap, our study evaluates the effectiveness of the “Marzhan Tis” mobile application, a gamified oral hygiene tool specifically designed for Kazakhstan children with ASD. The application includes a visual schedule of daily brushing routines, animated instructions with familiar characters, a reward system to reinforce task completion, and built-in reminders for caregivers. Additionally, this study aims to validate the reliability of a structured rating scale developed to assess adherence to oral hygiene practices in children with ASD. Together, these findings are intended to inform evidence-based interventions tailored to the needs of children with ASD in Kazakhstan and similar settings.

Thus, children with ASD face unique oral health challenges due to sensory, behavioral, and systemic barriers. While digital tools and visual supports show promise, there is a lack of ASD-specific interventions, especially in Kazakhstan. This study evaluates the impact of the gamified “Marzhan Tis” mobile application and validates a scale to assess oral hygiene adherence in children with ASD.

## Methods

### Study design

The study used a non-randomized cohort design, collecting data before and after the implementation of the mobile application to evaluate its effectiveness in improving toothbrushing skills among children with ASD. Participants were assigned to either the intervention group (IG) or the control group (CG) using a matched-group allocation approach. The groups were matched based on key variables, including age and caregiver involvement, to minimize confounding effects and ensure comparability.

### Participants

The study involved 90 children diagnosed with ASD, aged 3–18 years, who were enrolled in specialized rehabilitation centers in Almaty, Kazakhstan. Only children with Level 1 ASD, as defined by the fifth edition of the Diagnostic and Statistical Manual of Mental Disorders (DSM-5) were included. These children generally demonstrate mild impairments in social interaction and restricted behaviors but can follow basic verbal and visual instructions— making them suitable for app-based interventions. Children with Level 2 or Level 3 ASD were excluded, as their higher support needs would likely interfere with independent use of the mobile application.

Participants were further stratified into three age groups: 3–5, 6–11, and 12–18 years, to control for developmental differences. The intervention group used the “Marzhan Tis” mobile application for 1 month, which included visual prompts, structured routines, and gamified elements to promote oral hygiene. The control group received standard oral hygiene instructions without a mobile application.

The study was conducted in accordance with the Declaration of Helsinki and approved by the Local Ethics Committee of the S.D. Asfendiyarov Kazakh National Medical University (decision No. 8(114), dated 30.06.2021). Written informed consent was obtained from all participants’ parents or legal guardians prior to their inclusion in the study.

*Sample size calculation*: The required sample size was calculated using G*Power 3.1 software. Assuming a moderate effect size (*d* = 0.6) based on pilot data, *α* = 0.05, and power (1–β) = 0.80, the minimum sample size was estimated as 72 participants. To account for potential dropouts, 90 children were included in the study, which satisfied the statistical power requirements.

### Measures

All children underwent clinical assessments at baseline (T1) and after 1 month (T2). Oral hygiene status was evaluated using the Oral Hygiene Index (OHI), Simplified Oral Hygiene Index (OHI-S), and the approximal plaque index (API), while the condition of periodontal tissues was assessed with the papillary–marginal–attached index (PMA). OHI was assigned a numerical value: Good = 0, Satisfactory = 1, Unsatisfactory = 2, Poor = 3, And Very poor = 4. Hygiene indices were determined at T1 and T2 stages, allowing the evaluation of changes in oral health status after using the mobile application “Marzhan Tis”

To assess the impact of the intervention, both behavioral and clinical indicators of oral hygiene were evaluated using validated tools. A modified Oral Hygiene Assessment Scale—10 items (OHAS-10) and the standard Oral Hygiene Assessment Scale (OHAS) were applied to provide a comprehensive understanding of each participant’s hygiene practices and oral health status. The OHAS-10 was used to evaluate oral hygiene adherence across ten key domains, including frequency of brushing, caregiver assistance, use of adjunctive hygiene aids, behavioral cooperation, and familiarity with hygiene routines. Each item is scored on a 4-point Likert scale (0–3), where higher scores reflect poorer adherence to hygiene practices.

In parallel, the standard OHAS was used to clinically assess oral hygiene status through visual inspection of plaque accumulation and gingival inflammation on specific index teeth or surfaces. Scoring was based on the following criteria: Score 0—Excellent hygiene: no visible plaque or gingival inflammation; Score 1—Good hygiene: minimal plaque localized at the gingival margin, with slight gingival redness; Score 2—Fair hygiene: moderate plaque accumulation, noticeable gingival redness, and mild swelling; Score 3—Poor hygiene: heavy plaque accumulation, widespread gingival inflammation, and bleeding upon probing. Interpretation of scores was as follows: 0–1 indicates satisfactory oral hygiene; 2–3 suggests a need for improved oral hygiene practices or professional intervention.

To objectively assess plaque levels and enamel condition, the Qscan Plus (Qscan Plus, DP MediTech, Republic of Korea) diagnostic device was used. This device is equipped with four blue light LEDs and a special mirror screen that reflects the condition of the patient’s oral cavity. It enables safe and accurate assessment of tooth enamel using fluorescence, allowing for the detection of plaque, tartar deposits, and enamel micro-damage.

### Intervention group procedures

Participants in the intervention group attended a 30-min orientation session conducted by a pediatric dentist and a research assistant. During the session, caregivers received verbal and printed instructions on app usage. Children observed a live demonstration and practiced one brushing session using the app. Caregivers were encouraged to supervise daily use at home over the one-month intervention period.

### Control group procedures

Participants in the control group received standard oral hygiene education through an illustrated leaflet and a short verbal session (10–15 min) conducted by the same dentist who worked with the intervention group. The leaflet outlined recommended brushing techniques and hygiene practices, ensuring consistent baseline knowledge across both groups.

### Clinical assessment

All clinical assessments were performed by two calibrated pediatric dentists. Intra- and inter-examiner reliability were tested prior to data collection using Cohen’s kappa statistics on 20 randomly selected cases. Agreement levels were high, with inter-examiner kappa values ranging from 0.82 to 0.89 and intra-examiner values exceeding 0.90.

### Survey on children’s oral hygiene practices

To comprehensively evaluate parental awareness and engagement in their children’s oral hygiene, a structured 15-item questionnaire was developed and distributed among caregivers of study participants. The survey was designed to capture both quantitative and qualitative data, ensuring a well-rounded understanding of the challenges and behavioral patterns associated with oral hygiene maintenance in children with ASD.

The questionnaire included demographic information, gathering data on caregivers’ age, gender, and educational background. It assessed the level of parental involvement in daily toothbrushing routines by distinguishing between supervised and independent brushing, as well as inquiring about the frequency and extent of that involvement. The questionnaire explored perceived challenges in maintaining a child’s oral hygiene, such as sensory sensitivities, behavioral resistance, and motor skill deficits. It measured caregivers’ awareness and knowledge of proper oral hygiene practices—covering brushing techniques, the use of additional products like mouthwash and dental floss, and the frequency of dental visits. Parents were invited to evaluate the perceived effectiveness of the “Marzhan Tis” application in improving their child’s adherence to brushing and overall motivation. Lastly, the questionnaire recorded any behavioral changes in the child’s oral hygiene habits that caregivers observed following the intervention period.

The questionnaire began by gathering general information about each child, including age, specific diagnosis, and the level of support needed. It then asked caregivers about their previous experience with mobile applications to assess familiarity and comfort with digital tools. A key section evaluated the child’s hygiene skills both before and after using the app, enabling a direct comparison of any improvements. Finally, parents were invited to rate their satisfaction with the app’s functionality and user interface, providing feedback on usability and design.

Application usability was assessed through five Likert scale items embedded in the caregiver questionnaire. Each item (ease of use, clarity, child engagement) was scored from 1 (very poor) to 5 (excellent). The sum of these responses formed the total usability score (range: 5–25), with higher scores indicating a more favorable use experience.

The content validity of the caregiver questionnaire was assessed by two experts in pediatric dentistry and behavioral science using the Index of Item-Objective Congruence (IOC). Items with IOC values below 0.6 were revised or removed to ensure clarity and relevance.

### “Marzhan Tis” mobile application

**Fig. 1:**
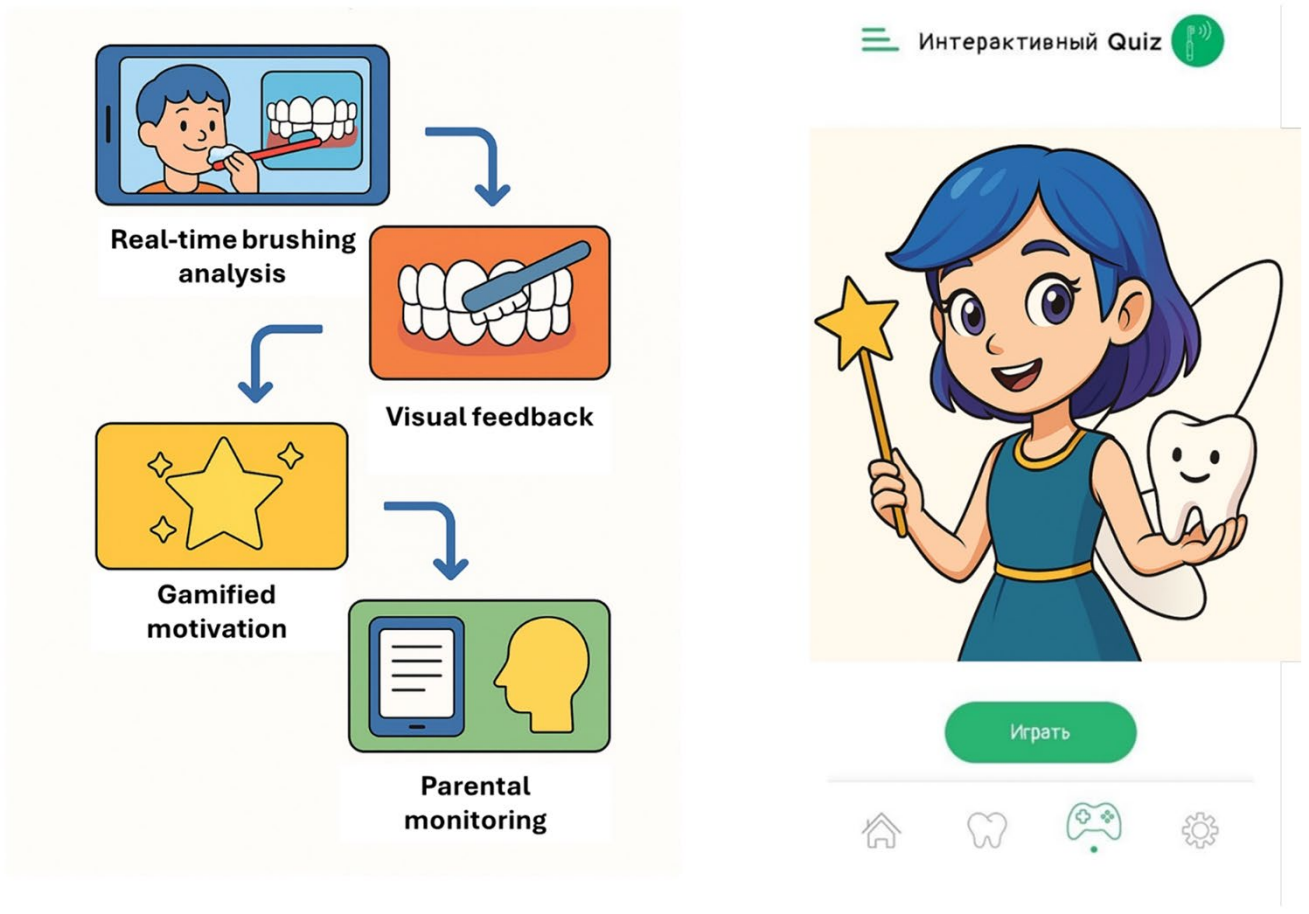
User interface of the “Marzhan Tis” mobile application

The application was specifically designed to support the development of oral hygiene skills in children with ASD (Fig. 1). The app incorporates evidence-based behavioral strategies, including video modeling, visual prompts, and gamification elements, to engage children and promote routine adherence. Customizable features allow caregivers to tailor sound levels, visual feedback, and brushing duration to accommodate sensory sensitivities common in ASD. The app provides interactive tutorials on proper brushing techniques and uses reminder notifications to reinforce daily hygiene habits. A built-in tracking system enables caregivers to monitor progress over time. Developed with input from pediatric dentists, special education professionals, and caregivers, “Marzhan Tis” offers a user-friendly interface suitable for children aged 3–18 years. Its design reflects a culturally adapted approach to oral health education, making it a relevant tool for use in Kazakhstan and similar healthcare settings.

The brushing technique taught via the application varied by age group. Children aged 3–11 years were taught the modified Fones technique using circular motions for simplicity. Adolescents aged 12–18 years received instructions in the horizontal scrub technique, reflecting greater manual dexterity. Age-appropriate animated videos and voice-over cues were used to guide children through each step of brushing all tooth surfaces.

### Statistical analysis

Data were analyzed using SPSS Version 26.0 (IBM Corp., Armonk, NY, USA). Descriptive statistics were used to summarize demographic characteristics and oral hygiene scores. The Shapiro–Wilk test was applied to assess the normality of data distribution. For within-group comparisons before and after the intervention, the Wilcoxon signed-rank test was used due to non-parametric distribution of the OHI scores. Between-group comparison of oral hygiene indices (OHI-S, API, PMA) were performed using the Mann–Whitney *U* test. Categorical data, including levels of hygiene skill and independence, were analysed using the Chi-square test (*χ*^2^). To explore the relationship between app usability and improvements in oral hygiene skills, Pearson’s correlation coefficient (*r*) was calculated. Statistical significance was set at *p* <0.05.

## Results

A total of 90 children diagnosed with ASD, aged 3–18 years, were enrolled in the study. An age-stratified analysis showed significant improvements in oral hygiene status across all age groups following the intervention. In the 3–5 age group, “Good” hygiene ratings increased from 3 to 15 children, while “Poor” ratings dropped from 7 to 2. Similar trends were seen in the 6–11 and 12–18 age groups, with “Good” hygiene rising and “Poor” hygiene decreasing notably (Table 1).

**Table 1:** Distribution of oral hygiene status before and after the intervention by age group (*n* = 90)

Age group (years)	Time point	Good	Satisfactory	Unsatisfactory	Poor	Very poor
3–5	Baseline	3	7	10	7	3
	1 month	15	10	3	2	0
6–11	Baseline	5	8	10	5	2
	1 month	12	10	5	2	1
12–18	Baseline	5	7	10	8	4
	1 month	10	8	6	4	2

### Oral Hygiene Index improvements

**Fig. 2:**
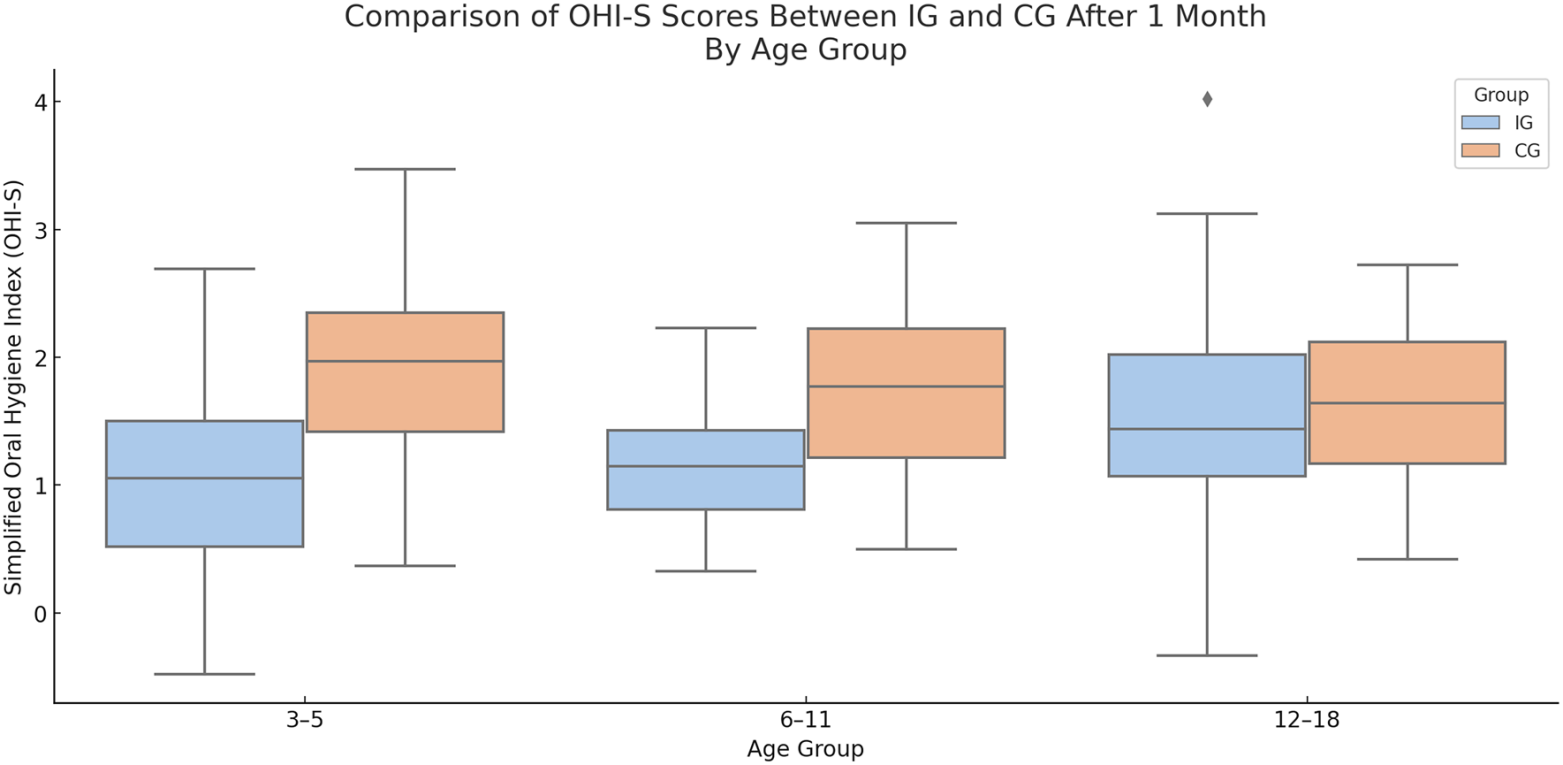
Box-and-whisker plot comparing OHI-S scores between intervention (IG) and control (CG) groups after 1 month, by age group

Significant improvements in oral hygiene were observed in the IG across all age categories. The OHI-S scores decreased by 28.2–51.7% in the IG, compared to only 9.4–9.5% in the CG. The greatest reduction was observed in the 3–5 year age group of the IG, where OHI-S dropped from 2.07 ± 0.84 to 1.00 ± 0.73 (*p* = 0.001; Cohen’s *d* = 1.38). In contrast, the same age group in the CG showed smaller change (from 2.05 to 1.85, *p* = 0.049) (Table 2). These visualizations (Fig. 2) highlighted the differential impact of the mobile application on oral hygiene outcomes.

**Table 2:** Summary comparison of oral hygiene indicators in the control (CG) and intervention groups (IG) before and after 1-month intervention

Index	Group		T1 (baseline) Mean ± SD	T2 (1 month) Mean ± SD	% Change	*p*-value	
						Within-group (Wilcoxon)	Between- group (U test)
OHAS	CG		62.5 ± 3.8	65.8 ± 4.2	+ 5.3%	0.05	*p* < 0.0001
	IG		63.1 ± 3.5	85.3 ± 2.1	+ 35.2%	0.001	
OHI	CG		2.02 ± 0.85	1.82 ± 0.78	− 10%	0.045	*p* = 0.038
	IG		2.11 ± 0.88	1.48 ± 0.75	− 30%	0.001	
OHAS-10	CG		72.4 ± 3.2	75.2 ± 3.5	+ 3.9%	0.040	*p* < 0.0001
	IG		71.8 ± 3.5	85.3 ± 2.1	+ 18.8%	0.001	

	Age, group		T1 Mean ± SD	T2 Mean ± SD	Reduction % (95% CI)	*p*-value		Effect size (Cohen’s d)
						Within group (Wilcoxon)	Between group (U test)	
OHI-S	3–5 years	IG	2.07 ± 0.84	1.00 ± 0.73	51.7 (46.2–57.1)	0.001	*p* < 0.0001	1.38 (large)
		CG	2.05 ± 0.82	1.85 ± 0.76	9.8 (7.1–12.5)	0.049		0.24 (small)
	6–11 years	IG	1.93 ± 0.78	1.27 ± 0.65	34.2 (29.8–38.6)	0.003	*p* = 0.004	0.92 (large)
		CG	1.91 ± 0.75	1.73 ± 0.72	9.4 (6.6–12.2)	0.050		0.23 (small)
	12–18 years	IG	2.13 ± 0.92	1.53 ± 0.81	28.2 (23.4–33.0)	0.02	*p* = 0.292	0.71 (medium)
		CG	2.10 ± 0.90	1.90 ± 0.82	9.5 (6.5–12.5)	0.048		0.22 (small)
API	3–5 years	IG	76.4 ± 5.1	33.2 ± 4.9	56.5 (53.1–59.9)	0.001	*p* < 0.001	8.73 (huge)
		CG	76.0 ± 5.2	68.3 ± 5.4	10.1 (7.2–13.0)	0.046		1.44 (large)
	6–11 years	IG	75.1 ± 4.8	31.8 ± 4.6	57.7 (54.3–61.1)	0.001	*p* < 0.001	9.24 (huge)
		CG	74.9 ± 4.7	67.1 ± 5.1	10.4 (7.6–13.2)	0.045		1.46 (large)
	12–18 years	IG	75.9 ± 5.3	30.7 ± 4.5	59.6 (56.4–62.8)	0.001	*p* < 0.001	9.48 (huge)
		CG	75.6 ± 5.0	68.0 ± 5.3	10.0 (6.8–13.2)	0.044		1.42 (large)
PMA	3–5 years	IG	43.2 ± 6.0	19.5 ± 5.1	54.9 (51.0–58.8)	0.001	*p* < 0.001	4.32 (huge)
		CG	42.9 ± 5.9	39.0 ± 5.7	9.1 (5.9–12.3)	0.048		0.72 (medium)
	6–11 years	IG	42.8 ± 5.8	18.1 ± 5.0	57.7 (54.1–61.3)	0.001	*p* < 0.001	4.67 (huge)
		CG	42.5 ± 5.7	38.4 ± 5.5	9.6 (6.2–13.0)	0.047		0.75 (medium)
	12–18 years	IG	41.5 ± 6.3	17.3 ± 5.5	58.3 (54.5–62.1)	0.001	*p* < 0.001	4.21 (huge)
		CG	41.3 ± 6.1	37.2 ± 5.8	9.9 (6.6–13.2)	0.045		0.68 (medium)

*OHAS* oral hygiene assessment score, *OHI-S* Oral Hygiene Index-Simplified, *OHAS-10* Oral Hygiene Assessment Scale-10 items, *API* approximal plaque index, *PMA* papillary–marginal–attached index, *IG* intervention group, *CG* control group

### Plaque and gingival status

The API and PMA improved markedly in the IG, with average reductions of 56.5–59.6% and 54.9–58.3%, respectively (*p* < 0,001). In the CG, only modest improvements (approximately 9–10%) were recorded. These differences were consistent across all age groups (Table 2).

## Oral hygiene adherence

The OHAS-10 increased significantly in the IG, from 71.8 ± 3.5 to 85.3 ± 2.1 (*p* = 0.001), reflecting enhanced behavioral compliance. The CG showed a smaller increase from 72.4 to 75.2 (*p* = 0.04) (Table 2).

### Survey results

Parental questionnaires (*n* = 90) indicated that sensory sensitivity (70%) and behavioral resistance (50%) were the most common obstacles to effective oral care. Other challenges included time constraints (25%) and limited access to specialized dental services (18%). While only 40% of caregivers demonstrated sufficient brushing techniques knowledge, 60% (*n* = 54, 95% CI 49.9–70.1%) expressed interest in additional education. The “Marzhan Tis” app received a positive usability rating from 85% of respondents (95% CI 76.9–91.9%). Its most valued features included visual prompts (70%), video tutorials (60%), gamification (50%), and reminders (45%). Improved cooperation during brushing was reported by 75% (*n* = 68, 95% CI 65.4–83.5%) of parents, and 40% (*n* = 36, 95% CI 29.9–50.1%) observed visible reductions in plaque. These results underscore the necessity of structured parental involvement in oral hygiene maintenance and highlight the promising role of digital health tools in fostering sustainable behavioral changes in children with ASD.

### Skill development and independence

The majority of caregivers (65%, *n* = 59) reported assisting their children with daily brushing (95% CI 54.6–74.3%), while 35% (*n* = 31) indicated that their children brushed independently with minimal supervision (95% CI 24.6–44.3%). Only 10% (*n* = 9) of children in the IG and 12% (*n* = 11) in the CG were able to brush independently. After 1 month, significant improvement was observed in the IG: the number of independently brushing children increased to 40% (*n* = 36), and those requiring full assistance decreased from 20 to 5% (*χ*^2^ = 28.7, *p* < 0.001). In contrast, the CG showed only a modest improvement: independent brushing rose slightly to 18% (*n* = 16), and the proportion of children needing full assistance declined from 22 to 15% (*χ*^2^ = 6.1, *p* = 0.047). Hygiene index category analysis confirmed a shift toward better performance in the IG. Prior to the intervention, 40% of IG participants (*n* = 36) struggled with hygiene routines and 20% (*n* = 18) were completely unable to perform them. Post-intervention, these numbers dropped to 20% and 5%, respectively. Meanwhile, in the CG, only minor changes were noted in these categories from poor to satisfactory or good hygiene levels.

These findings highlight the superior impact of the “Marzhan Tis” application in fostering hygiene skill acquisition and independence compared to standard oral hygiene instruction.

**Fig. 3:**
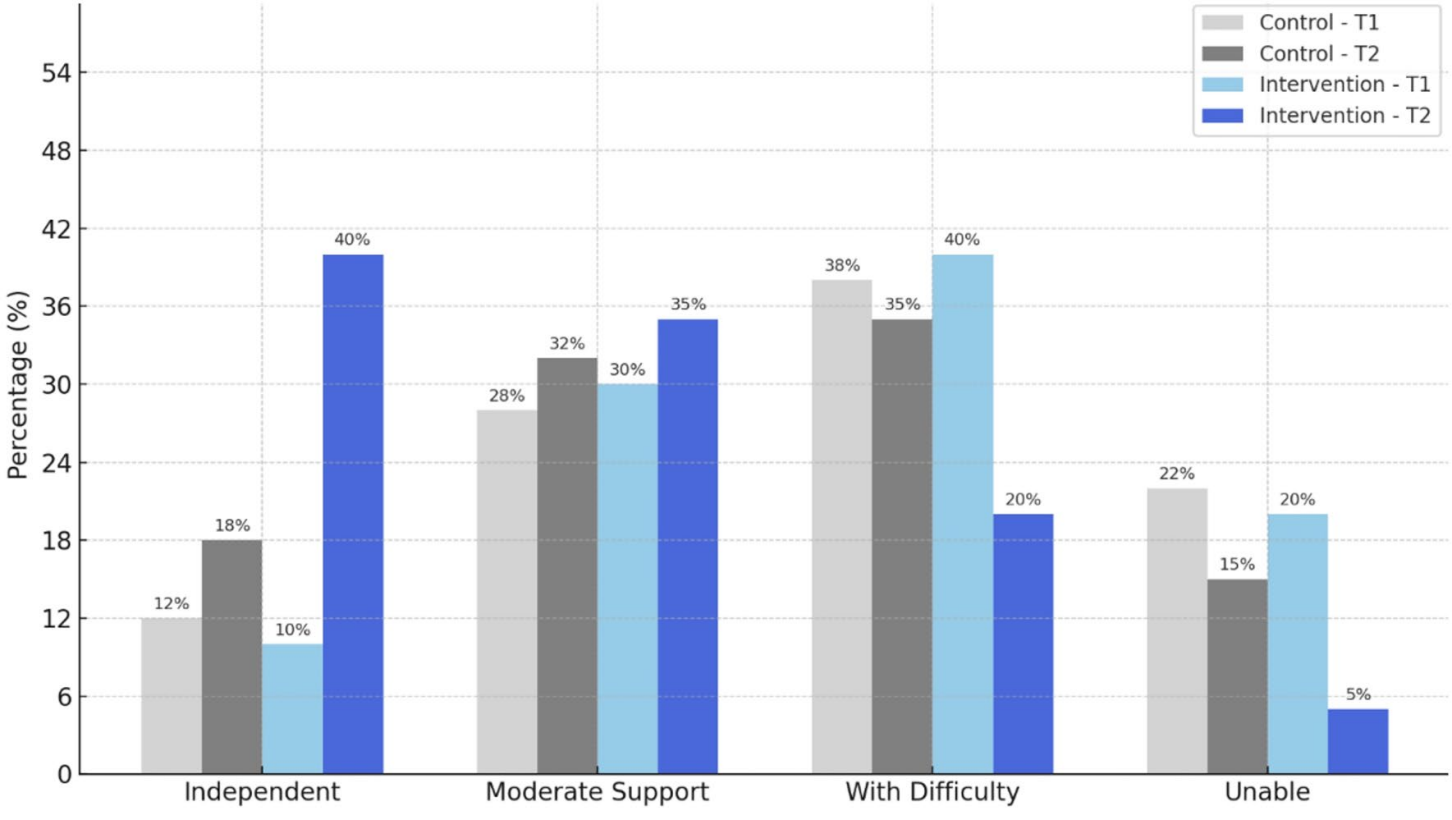
Change in hygiene independence by group and time point

Prior to the intervention, only 10% of children (*n* = 9) were able to carry out hygiene routines easily, while 30% (*n* = 27) performed them with moderate assistance. A substantial proportion of children—40% (*n* = 36) were reported to manage hygiene with difficulty, and 20% (*n* = 18) were unable to perform these tasks at all. Following the 6-week use of the app, the proportion of children who could independently and easily perform hygiene routines rose to 40% (*n* = 36). Those who managed moderately increased to 35% (*n* = 31), while the number of children performing with difficulty dropped to 20% (*n* = 18). Notably, only 5% (*n* = 5) remained unable to perform hygiene tasks (Fig. 3). This shift reflects a clear improvement in functional independence and supports the app’s role in enhancing routine hygiene behavior among children with ASD.

### Usability and outcome correlation

**Fig. 4:**
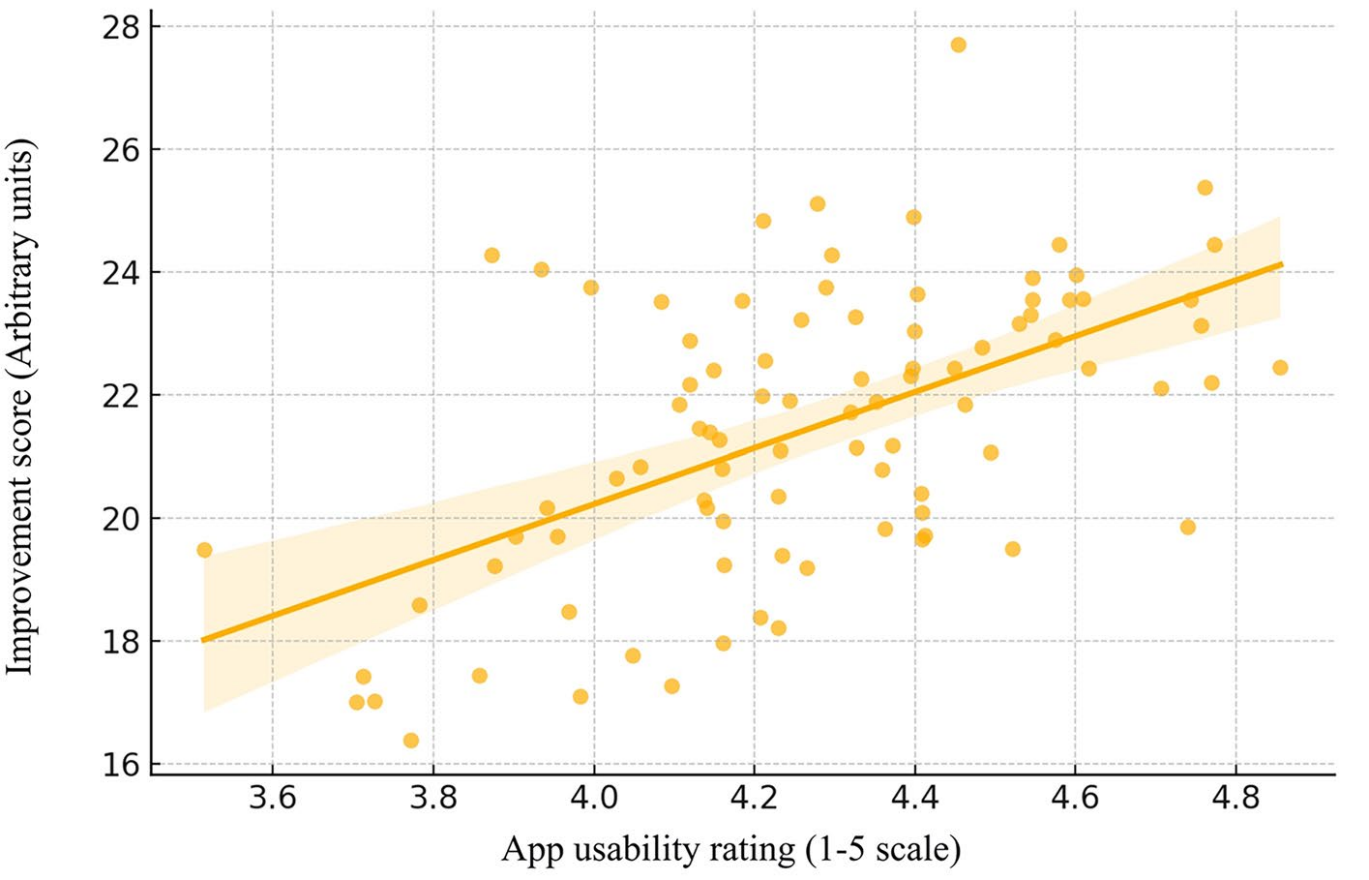
Scatter plot depicting the correlation between app usability ratings and hygiene improvement scores

The mean usability rating was 4.3 out of 5. A moderate to strong positive correlation was identified between usability scores and improvement in hygiene skills (*r* = 0.65, *p* = 0.01), suggesting that intuitive, accessible design contributes meaningfully to behavioral change (Fig. 4).

## Discussion

This study demonstrated that the “Marzhan Tis*”* mobile application significantly improved oral hygiene skills among children with autism spectrum disorder (ASD) Level 1. These findings are consistent with prior research indicating that children with ASD frequently exhibit poor oral health and face substantial challenges in maintaining independent hygiene routines (El Khatib et al. 2014; Jaber 2011). For example, El Khatib et al. (2014) and Jaber (2011) reported high levels of untreated caries and behavioral difficulties during oral care, underscoring the critical need for targeted interventions. While the application demonstrated significant improvements in oral hygiene skills among children with ASD Level 1, it may not be applicable to all ASD patients, particularly those with more severe forms requiring individualized behavioral support.

Before the intervention, only 10% of participants were able to perform oral hygiene tasks independently, a finding that mirrors previous reports in which the majority of autistic children struggled with basic hygiene behaviors. Following four weeks of application use, this proportion increased significantly to 40%, while the number of children experiencing difficulty declined from 60 to 25% (*χ*^2^ = 28.7, *p* < 0.001). These improvements were statistically significant and were not observed in the control group, reinforcing the intervention’s specific effect. The results align with those of Suhaib et al. and Zerman et al., who emphasized the benefits of structured support in improving oral health outcomes in this population (Zerman et al. 2022; Suhaib et al. 2019).

The most notable improvements were observed in toothbrushing skills, a task widely acknowledged as particularly challenging due to sensory sensitivities and behavioral resistance (Chauhan et al. 2025). This is consistent with the findings of Chauhan et al. (2025), who described autistic children perceiving toothbrushing as an “explosion in the mouth.” Unlike standard apps, the “Marzhan Tis” incorporated customizable sensory adaptations, including adjustable sound levels, neutral visual aesthetics, and smooth-edged graphics, to help mitigate sensory overload.

A moderate to strong positive correlation (*r* = 0.65, *p* < 0.01) is observed between app usability ratings and hygiene improvement, confirming previous findings that visually structured and user-friendly tools are especially effective for children with ASD (Bondioli et al. 2024; Liu et al. 2023). These results are in line with prior evidence emphasizing the value of visual instruction and modeling strategies in educational interventions for this group (Liu et al. 2023).

When compared with the iPad-based toothbrushing program evaluated by Lopez Cazaux et al. (2019), “Marzhan Tis*”* yielded comparable gains in brushing frequency and technique. Both interventions highlight the essential role of caregiver involvement and stepwise instruction. However, unlike the iPad program, “Marzhan Tis*”* was designed with sensory sensitivities in mind, offering enhanced personalization that may further facilitate user engagement and task adherence.

In addition to behavioral outcomes, systemic access barriers were identified, with 18% of caregivers reporting limited access to specialized dental care. This echoes previous findings suggesting that access disparities remain a major obstacle to oral health for children with ASD (Salerno et al. 2025). In such contexts, mobile health technologies like “Marzhan Tis*”* may serve as scalable and accessible tools, especially in underserved or rural areas where specialized services are scarce.

Several limitations should be acknowledged. Although a control group was included, the relatively small sample size limits the generalizability of the results. Furthermore, reliance on caregiver-reported outcomes introduces the potential for subjective bias. Evaluators conducting clinical assessments were not blinded to group allocation, which may have introduced observational bias. Additionally, the non-randomized group allocation may have introduced selection bias despite the use of matched-group design. Future research should aim to conduct randomized controlled trials with larger, more diverse samples and incorporate objective clinical assessments. Longitudinal study designs are also recommended to evaluate the sustainability of behavioral improvements and the long-term impact of digital interventions on oral health in children with ASD. Future studies should enhance methodological rigor by employing randomized allocation and long-term follow-up to validate the observed differences between intervention and control groups.

## Conclusion

The use of the “Marzhan Tis” mobile application led to statistically significant improvements in oral hygiene skills, plaque reduction, and hygiene adherence among children with ASD Level 1, compared to the control group receiving standard care. The application may not be generalizable to all ASD patients. The most effective features included video tutorials, visual prompts, and gamification. A strong positive correlation between app usability and hygiene improvement underscores the importance of user-centered design.

Children in the intervention group showed a greater increase in independent hygiene performance and more pronounced improvements across all oral hygiene indices, particularly in the youngest age group (OHI improvement: 51.7%, *p* = 0.001). Oral hygiene adherence improved by 18.8% in the intervention group (*p* = 0.001), compared to minimal changes in the control group (*p* = 0.040). These findings support the app’s role as an effective and scalable tool for promoting oral health behaviors in children with ASD.

Future studies should focus on strengthening methodological rigor by employing randomized allocation, blinding of assessors, and long-term follow-up. Broader investigations across diverse populations are recommended to confirm generalizability and assess the sustainability of behavioral improvements over time.

## Data Availability

No datasets were generated or analyzed during the current study.
